# Radical Resection of Small Bowel Adenocarcinoma With Multiple Liver Metastases Following Neoadjuvant Chemotherapy: A Case Report

**DOI:** 10.7759/cureus.69776

**Published:** 2024-09-20

**Authors:** Kei Kobayashi, Kazunori Nojiri, Hirokazu Suwa, Kenichi Yoshida, Hidenobu Masui

**Affiliations:** 1 Department of Gastrointestinal Surgery, Yokosuka Kyosai Hospital, Yokosuka, JPN

**Keywords:** jejunum adenocarcinoma, liver metastases, neoadjuvant chemotherapy, small bowel adenocarcinoma, surgical resection

## Abstract

Small bowel adenocarcinoma (SBA) is a relatively rare disease that is difficult to detect in the early stages; therefore, it often has a poor prognosis. We present a rare case of SBA and multiple liver metastases in a patient who underwent curative resection combined with neoadjuvant chemotherapy (NAC). A 70-year-old woman presented to the emergency department complaining of abdominal pain and bloating. She was diagnosed with bowel obstruction due to a primary jejunal tumor and was admitted to the hospital. After further imaging and histopathological examination, the patient was diagnosed with primary jejunal adenocarcinoma with multiple liver metastases, all of which were considered resectable. Since she had developed bowel obstruction due to the primary tumor, jejunal resection with draining lymph node removal was initially performed. The remaining multiple liver metastases were treated with four courses of capecitabine and oxaliplatin (CAPEOX) with bevacizumab as NAC, followed by hepatectomy. After NAC, the patient underwent radical liver resection. Based on a pathological examination, the five liver tumors were all diagnosed as liver metastases from jejunal adenocarcinoma. Six months after the liver resection, a single recurrence was observed in segment V of the liver. Therefore, four courses of CAPEOX with bevacizumab were administered again as NAC, and liver resection was performed again. At the time of writing this report, she has survived for more than four years after the first surgery, with no apparent recurrence. This is a rare case of a patient who underwent radical resection of SBA with multiple liver metastases following CAPEOX and bevacizumab as NAC.

## Introduction

Primary small-bowel malignancies are relatively rare, accounting for approximately 3% of all gastrointestinal malignancies [1]. Small bowel adenocarcinoma (SBA) accounts for approximately 30% to 40% of all small bowel malignancies, with other common types being carcinoid tumors, sarcomas, and lymphomas [2]. Owing to its rarity and non-specific symptoms, patients with SBA are often diagnosed at an advanced stage, and their five-year overall survival (OS) rate is approximately 26% across all stages [3]. The standard treatment for localized SBA is surgical resection with en-bloc removal of the regional lymph nodes; however, the therapeutic strategy for SBA with distant metastases has not been fully established [4,5]. Although recent reports have shown the benefit of radical resection for SBA with distant metastases, systemic chemotherapy is considered the standard treatment for stage IV disease [5-9]. Thus, the indications for surgical resection and neoadjuvant chemotherapy (NAC) for metastatic disease are still unknown owing to the lack of appropriate randomized prospective trials. We herein present the rare case of a 70-year-old woman with multiple liver metastases from SBA who underwent curative resection combined with NAC.

## Case presentation

**Figure 1:**
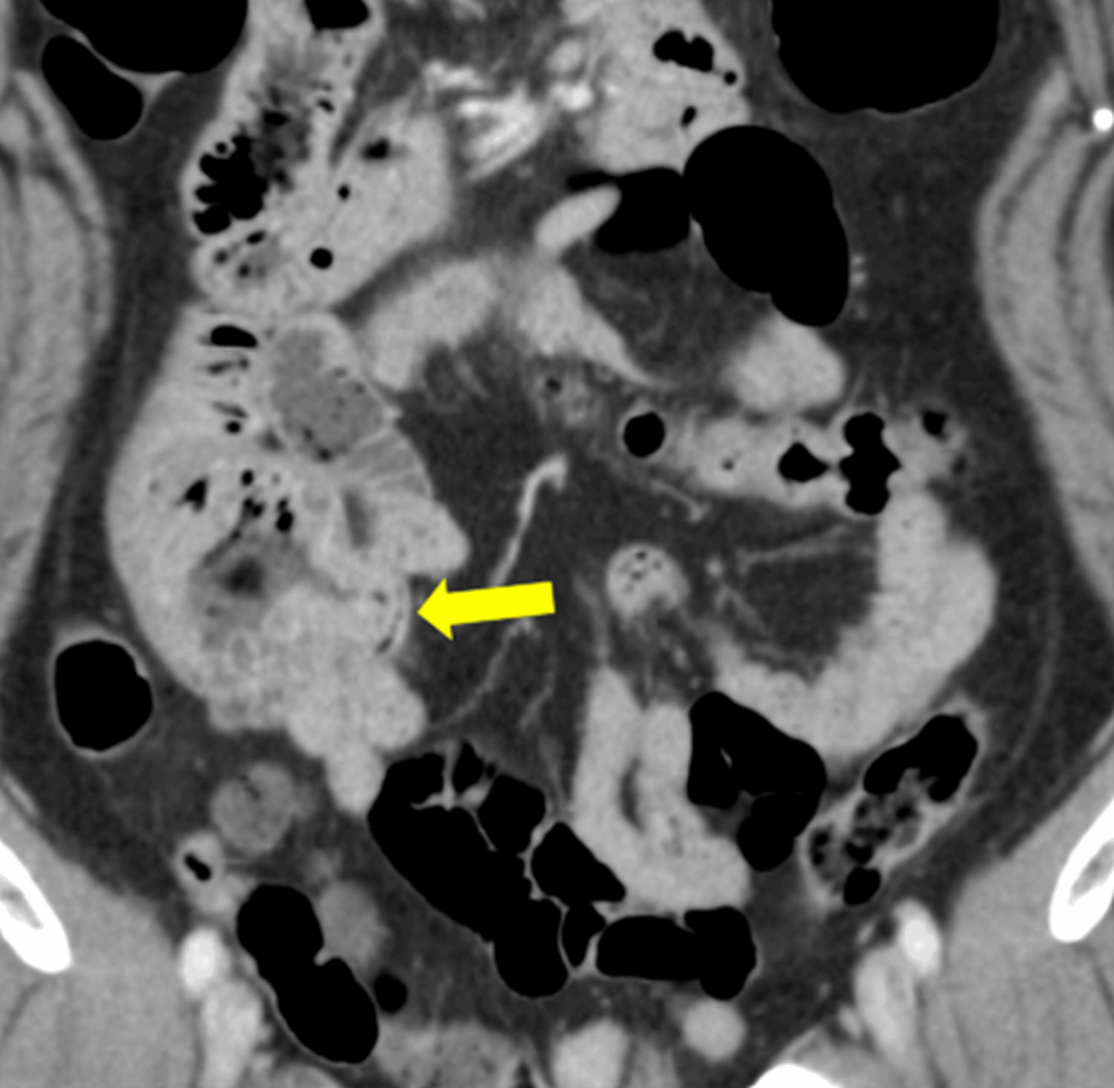
Abdominal CT Abdominal CT showed wall thickening of the jejunum. CT: computed tomography

A 70-year-old woman initially presented to the emergency department in June 2020 with abdominal pain and bloating. A physical examination revealed tympanic sounds and dull pain in the upper abdomen without rebound tenderness or rigidity. Abdominal contrast-enhanced CT showed total circumferential wall thickening in the jejunum and dilation of the duodenum and stomach (Figure 1).

**Figure 2:**
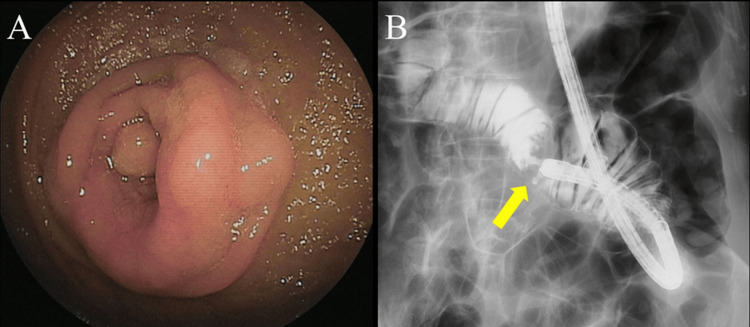
Small bowel endoscopy and radiograph A: Small bowel endoscopy revealed a localized-ulcerating tumor encircling the jejunum; B: Small bowel radiograph showing an apple core sign in the jejunum, 40 cm from the ligament of Treitz (arrow)

**Figure 3:**
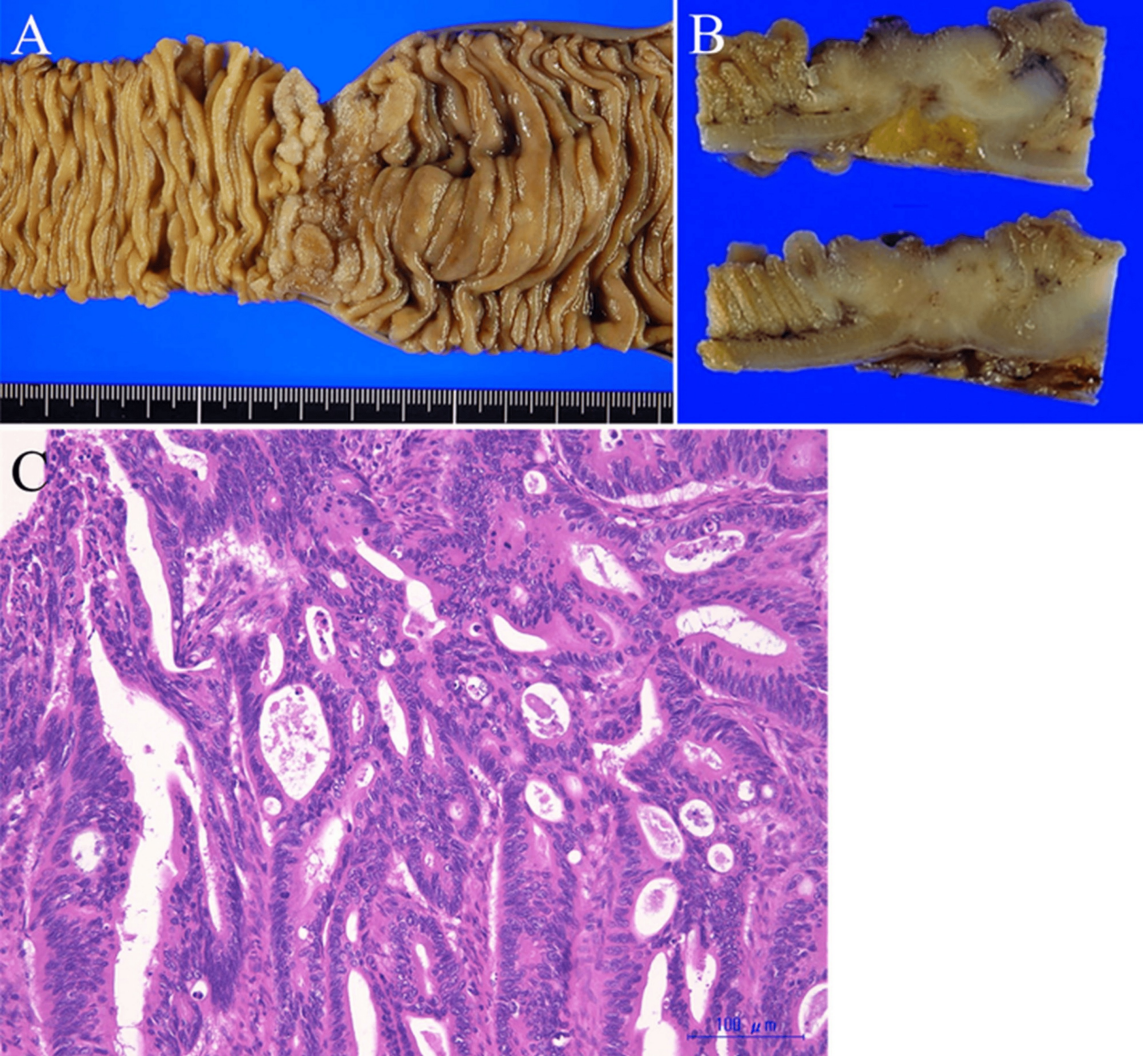
The resected specimen of the primary jejunal tumor A: Gross examination of the resected specimen of the primary jejunal tumor revealed localized ulceration; B: The cut surface of the tumor showed deeper invasion across the serosa of the jejunal wall; C: Hematoxylin-eosin staining The tumor was histologically diagnosed as well-differentiated adenocarcinoma.

The patient was suspected to have bowel obstruction caused by jejunal cancer and was referred to the gastroenterology department for further examination. A small bowel endoscopy revealed a localized ulcerative tumor encircling the jejunum, approximately 40 cm from the ligament of Treitz (Figures 2A, 2B). The tumor was pathologically diagnosed as a well-to-moderately differentiated adenocarcinoma via biopsy. Further examination revealed a total of five simultaneous liver metastases (A 7 mm tumor in segment IV, 14 mm and 8 mm tumors in segment V, and 6 mm and 4 mm tumors in segment VII) without obvious peritoneal dissemination or any other distant metastases. Finally, the patient was clinically diagnosed with primary stage IV jejunal adenocarcinoma. At the time of the diagnosis, both the primary tumor and distant metastases were considered to have been curatively resected. Since she had developed bowel obstruction due to the primary tumor, jejunal resection with draining lymph node removal was performed first in July 2020. In the operation, the tumor was located 40 cm from the ligament of Treitz, so the jejunum was divided at both the oral and anal sides with a 10 cm margin. The third jejunal branch, which supplies the tumor, was ligated at the first bifurcation, and the mesenteric lymph nodes were removed. The resected specimen showed a circumferential tumor with a histological type of moderately differentiated adenocarcinoma (Figures 3A-3C).

**Figure 4:**
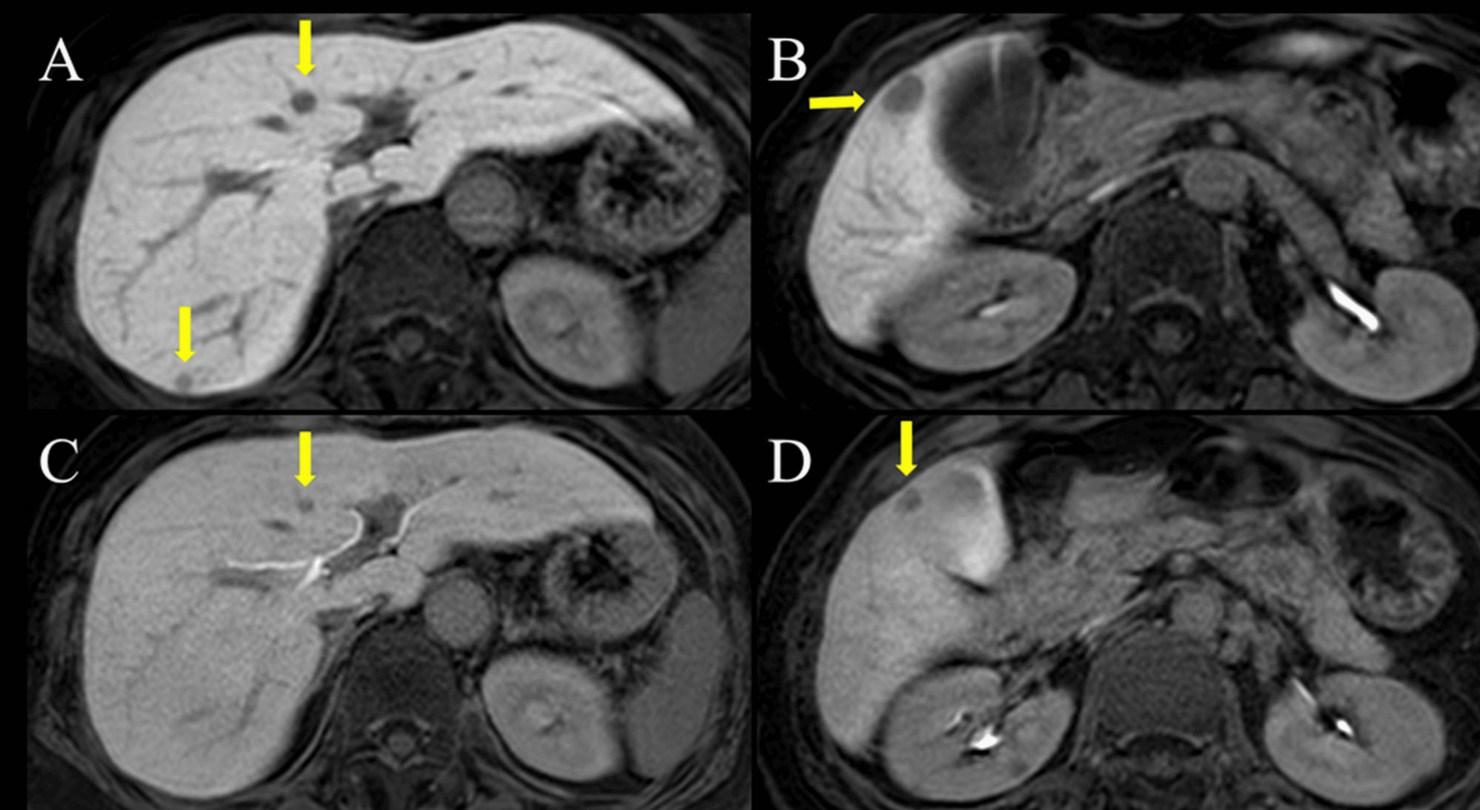
Abdominal MRI A, B: MRI showing liver tumors in segments IV, V, and VII before NAC (arrow); C, D: The tumors show a partial response after four courses of CAPEOX with bevacizumab (arrow). The tumor in segment VII was not clearly visible on the MRI slice. MRI: magnetic resonance imaging, CAPEOX: capecitabine and oxaliplatin

**Figure 5:**
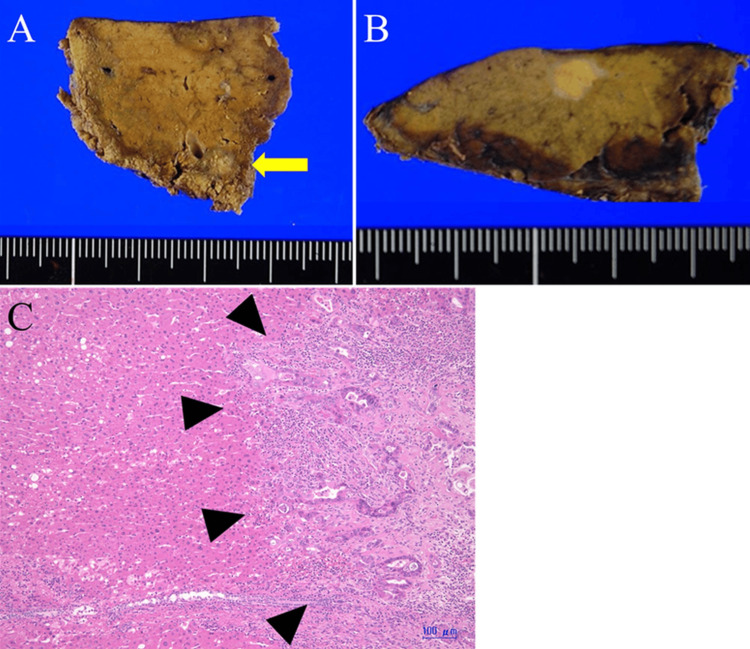
The resected specimens of the liver tumors A: Liver metastasis in segment IV (arrow); B: Liver metastasis in segment V; C: All tumors were histologically diagnosed as well-differentiated adenocarcinoma, which was derived from jejunal adenocarcinoma (arrowhead). The cut margins were negative.

There was one metastatic lymph node; therefore, the stage was pathologically diagnosed as T4N1 according to the eighth International Union Against Cancer (UICC) classification of malignant tumors. The remaining multiple liver metastases were treated with four courses of capecitabine and oxaliplatin (CAPEOX) with bevacizumab as NAC, followed by hepatectomy. After four courses of CAPEOX with bevacizumab, the liver metastases (7 mm in segment IV, 14 mm and 8 mm in segment V, and 6 mm and 4 mm in segment VII) showed a partial response (PR) according to the Response Evaluation Criteria in Solid Tumors (RECIST) guidelines (5 mm in segment IV, 9 mm and 5 mm in segment V, and 4 mm and 3 mm in segment VII) (Figures 4A-4D). In November 2020, she underwent liver resection, which included medial segmentectomy for tumors in segment IV and partial resection for tumors in segments V and VII, as planned. The patient was discharged on postoperative day 8 without complications. A pathological examination confirmed that the tumors were well to moderately differentiated adenocarcinomas originating from the primary jejunal cancer (Figures 5A-5C).

**Figure 6:**
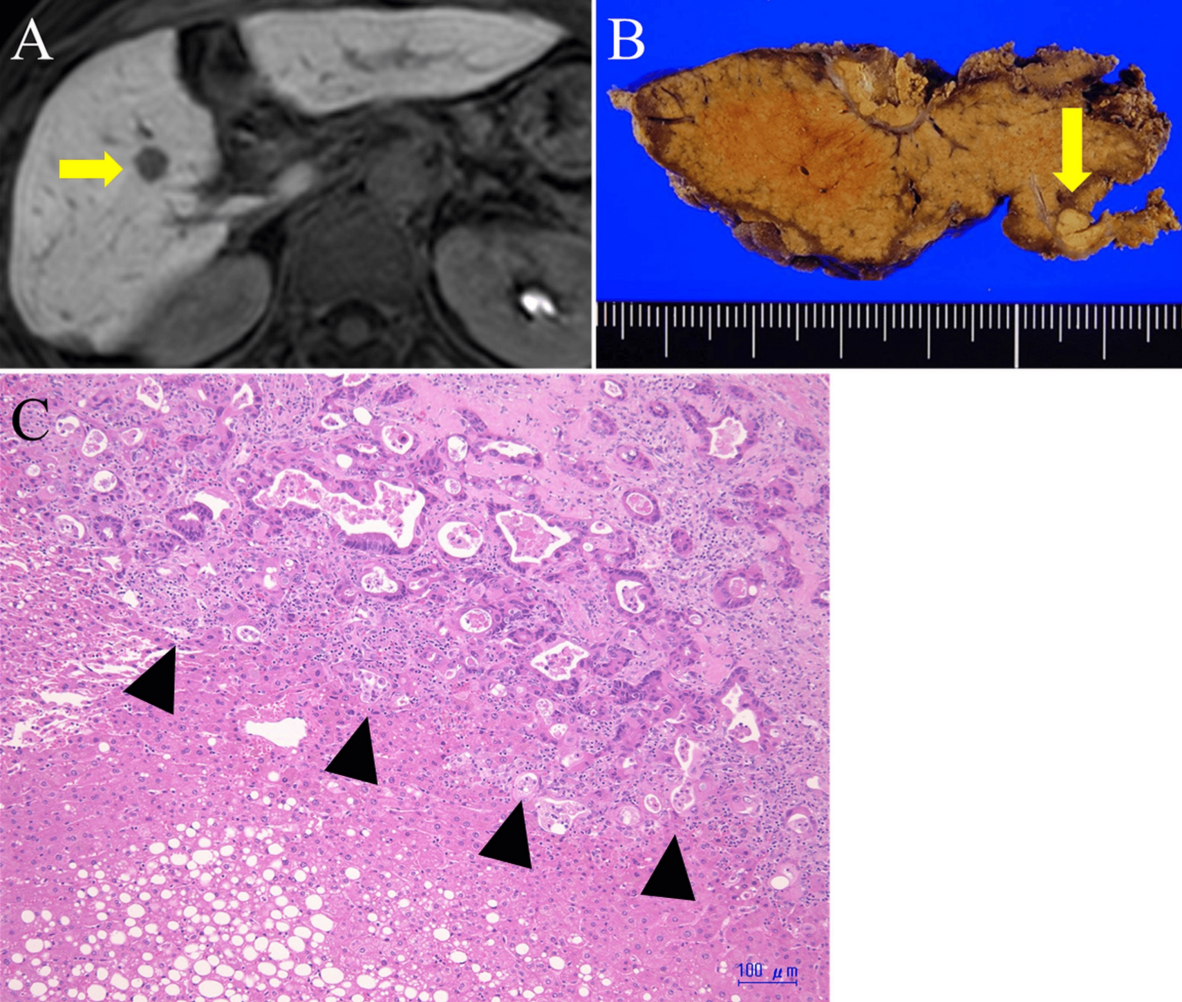
Single liver metastasis in segment V A: Abdominal MRI showed single liver metastasis in segment V (arrow); B: A resected specimen of the liver tumor (arrow); C: The tumor was histologically diagnosed as recurrent jejunal adenocarcinoma (arrowhead); MRI: magnetic resonance imaging

Although the patient had a high risk of recurrence, we did not administer adjuvant chemotherapy considering her condition and preferences. Therefore, the patient was closely monitored at our department. Six months after liver resection, a single 13 mm-sized tumor was detected in segment V of the liver (Figure 6A). Consequently, she underwent another liver resection after receiving four courses of CAPEOX with bevacizumab. The liver tumor was pathologically diagnosed as a recurrence of primary jejunal cancer (Figures 6B, 6C). After the last surgery, she showed no apparent recurrence. At the time of writing this report, she remains alive at more than four years after the first surgery.

## Discussion

In recent years, some reports have emphasized the beneficial effects of radical resection of SBA with distant metastases [6-9]. Rompteaux et al. reported a favorable prognosis, with a 3-year overall survival (OS) rate of 41% and a median survival time (MST) of 28.2 months, following radical metastasectomy in an analysis of 34 patients with stage IV SBA [7]. In addition, the authors revealed that poor differentiation, margin invasion, and lymphatic invasion in the primary tumor were associated with poor prognosis after metastasectomy. Therefore, they suggested that radical resection should be considered for SBA patients with well or moderately differentiated histology or when curative resection is expected. Moreover, Ye et al. showed that the combination of liver resection and chemotherapy may prolong the prognosis of SBA with liver metastasis [8]. As mentioned above, radical metasectomy for stage IV SBA should be considered depending on the individual case.

In our case, the patient developed bowel obstruction with a primary tumor at the time of the diagnosis. After further examination, she was diagnosed with jejunal adenocarcinoma with multiple liver metastases, and the histological type was well-to-moderately differentiated adenocarcinoma. Based on preoperative CT and MRI, both the primary tumor and liver metastases were considered resectable. Therefore, we performed radical resection of the tumors. Since the patient had developed bowel obstruction due to the primary tumor, we initially resected the primary jejunal tumor with lymph node dissection followed by liver resection. In this case, the lymph node was resected down to the origin of feeder vessels, in accordance with the National Comprehensive Cancer Network (NCCN) guidelines. Subsequently, to ensure curative resection of the liver metastases, NAC was performed with four courses of CAPEOX with bevacizumab prior to hepatectomy.

Although there are limited reports on chemotherapy for SBA, favorable outcomes have been reported for several regimens. Recently, the recommended chemotherapy regimens are FOLFOX (folinic acid + fluorouracil + oxaliplatin), CAPEOX, FOLFIRI (folinic acid + fluorouracil + irinotecan), or FORFIRINOX (folinic acid + fluorouracil + irinotecan + oxaliplatin) if patients tolerate chemotherapy [10-12]. Several phase II trials have been conducted in recent years, and FOLFOX and CAPEOX therapy have been reported to improve the prognosis [9,13,14]. Furthermore, the positive effect of combination therapy with bevacizumab or immune check inhibitors has also been reported [15,16]. According to these trials, the NCCN guidelines recommend FOLFOX and CAPEOX as first-line therapies. However, these regimens are used for palliative chemotherapy and not for perioperative chemotherapy. Therefore, the indications for surgical resection combined with perioperative chemotherapy for SBA liver metastases remain unknown. Since there is no established treatment strategy for SBA in Japan, we chose this regimen according to the therapeutic strategy for stage IV colorectal cancer. In our case, the use of bevacizumab, a humanized monoclonal antibody against vascular endothelial growth factor (VEGF), as NAC is controversial because of the risk of postoperative infectious complications. However, Scappaticci et al. reported that no increase in postoperative infectious complications was observed if at least four weeks were allowed between the end of chemotherapy and surgery [17]. Therefore, we performed liver resection at least four weeks after the last chemotherapy session. Considering the effect of shrinking tumors, we believe that bevacizumab in combination with CAPEOX is acceptable.

In the present case, the patient was treated with 4 courses of CAPEOX with bevacizumab for 3 months as NAC, and her liver metastases showed a PR with a 35% decrease in total tumor diameter. Consequently, liver resection was performed as planned, and all the tumors had negative pathological margins. At the time of writing this report, she has survived for more than four years after the first surgery. Similar to our case, Eigenbrod et al. reported a case in which a patient with multiple liver recurrences of SBA underwent conversion surgery after long-term FOLFOX therapy and had extended survival [18].

To our knowledge, this is the first report of the use of CAPEOX with bevacizumab as NAC for SBA with multiple liver metastases and long-term survival. However, whether surgical resection combined with perioperative treatment is effective for SBA with liver metastasis remains unclear. The accumulation of further cases is important to establish a therapeutic strategy for SBA with liver metastases.

## Conclusions

In the present case, the patient had advanced SBA with multiple liver metastases at the time of diagnosis. After four courses of CAPEOX with bevacizumab, we performed a curative resection of the liver metastases, and the patient has survived for more than four years following the initial surgery.

Although the beneficial effects of radical resection for liver metastases of SBA have been reported, the treatment strategy remains controversial. Combining NAC may contribute to increasing the possibility of achieving curative resection, making it a potentially effective treatment option for such cases.
